# Severe coagulopathy following snakebite envenomation and challenges in management: a report of two pediatric cases

**DOI:** 10.11604/pamj.2026.53.85.51351

**Published:** 2026-02-13

**Authors:** Émilie Ann Yansouni, Anna Monica Voia, Armand Kouassi Kakpo

**Affiliations:** 1 Faculté de Médecine, Université de Montréal, 2900 Édouard-Montpetit Blvd, Montréal, Québec, H3T 1J4, Canada,; 2 Pediatrics Service of *L'Abbraccio*, Hospital of Sokponta, Glazoué, Republic of Benin

**Keywords:** Snakebite, coagulopathy, antivenom, neglected tropical disease, case report

## Abstract

Snakebite envenomation (SBE) remains a major neglected tropical disease in sub-Saharan Africa, with West Africa accounting for a substantial proportion of global cases. In Benin, the true impact of SBE is underestimated due to limited epidemiological surveillance, delayed healthcare access, and limited availability of adequate urgent care. We report two fatal pediatric cases of severe Viperidae snakebite envenomation in children aged three and twelve years old managed at a rural hospital in Benin. Both patients died during hospitalization after developing severe coagulopathy despite antivenom therapy, blood transfusions, and supportive care. These cases illustrate the severity of hemorrhagic complications associated with SBE and, most importantly, highlight key contributors to poor clinical outcomes in low-resource settings. Diagnostic uncertainty and resource constraints further complicate the management of suspected complications such as compartment syndrome and intracranial bleeding. SBE remains a critical yet underrecognized cause of pediatric mortality in rural Benin. It would be essential to strengthen public health policies, improve access to effective antivenoms, and implement context-adapted management strategies to reduce SBE-related mortality in sub-Saharan Africa.

## Introduction

Snakebite envenomations (SBE) are a major public health challenge in West Africa, and this region accounts for approximately 20% of cases reported worldwide [1]. An estimated 10,000 to 30,000 deaths per year in Africa are attributable to SBE [2], and it is now recognized by the World Health Organization (WHO) as a neglected tropical disease. Crucially, gaps in epidemiological surveillance complicate both research and the effective allocation of resources that are required to prevent deaths. In Benin, the number of snakebites increases between March and August, before the rainy season, coinciding with the preparation and cultivation of fields. Although the country's snake fauna is not fully documented, over 90% of cases are caused by vipers such as *Echis ocellatus, Echis leucogaster, Bitis arietans*, and *Causus maculatus* [3]. Frequently observed complications include hemorrhage, coagulopathy, and severe kidney damage [1,4]. However, between 10% and 90% of bites from venomous snakes result in no clinical evidence of envenomation [4]. Timely management is hindered by the long distances to equipped health centers, putting rural populations at the highest risk of poor outcomes. Severe SBE outcomes often result from treatment delays, either from the use of ineffective traditional therapies before seeking care or from a lack of accessibility to effective antivenoms [4,5].

This case report and literature review sought to highlight research perspectives and strategies for better management of this serious health hazard affecting low-income communities in intertropical and subtropical regions. A literature search performed in multidisciplinary electronic databases showed that the prevalence of SBE is high in Benin, but the incidences, associated morbidities, and mortalities are greatly underestimated. Therefore, it would be imperative to revitalize the snakebite reporting system in order to have better epidemiological data and to develop a sustainable national strategy for the control and management of SBE. In this report, we present two fatal cases of SBE in rural Benin, highlighting the clinical severity and systemic complications associated with SBE. This case report underscores the urgent need for context-specific strategies to prevent mortality and novel reporting systems.

## Patient and observation

### Patient 1

**Patient information:** in April 2025, a three-year-old boy was brought to *L'Abbraccio* Hospital, in the town of Glazoué in Benin, after being bitten by a snake of the *Viperidae* family three days earlier. The patient had initially presented to another hospital before being referred. Upon admission, the patient presented with altered consciousness, edema, bleeding of his right upper limb, and vomiting.

**Clinical findings:** examination revealed severe palmar-plantar pallor, edema extending to the ipsilateral hemithorax, and significant limitation of range of motion in the right upper limb. Ruptured blisters were observed in the elbow crease and on the right hand.

**Timeline of current episode:** despite the administration of packed red blood cells (PRBC), fresh frozen plasma (FFP), and antivenom (Inoserp PAN-AFRICA (IPA) AVS, manufactured by Inosan Biopharma), the patient's condition deteriorated in the following hours. Tea-coloured urine was observed in the diaper. Prolonged bleeding developed at the bite site, suggestive of disseminated intravascular coagulation (DIC). The right upper limb grew increasingly tense and the fingers fixed in flexion, compatible with Volkmann's contracture seen in compartment syndrome. The patient also had two episodes of rectal bleeding. The next day, despite additional transfusions, the child developed shock and respiratory distress (Table 1).

**Table 1:** baseline characteristics of cases at the time of admission

	Case 1		Case 2		
Parameters	Patient values	Reference values	Patient values		Reference values
Hemoglobin (g/dL)	2.7	10.5-14.5	9.4		10.5-14.5
Platelets (G/I)	86	150-400	292		150-400
Leukocytes (G/I)	15.56	3-13	9.05		3-13
Neutrophils (G/I)	9.5	2-4	6.6		2-4
Lymphocytes (G/I)	4.7	0.5-0.8	1.2		0.5-0.8
aPTT (seconds)	41	25-26	39		25-26
PT/fibrinogen	N/A				
Blood pressure (mmHg)	97/56			121/67	
Axillary temperature (°C)	38			37.7	
Heart rate (beats per minute)	160			120	
Respiratory rate (breaths per minute)	62 (with O_2_)			20	
SpO_2_ (%)	94			100	
Capillary blood glucose (g/L)	4.1			1.59	

APTT: activated partial thromboplastin time, PT: prothrombin time

**Diagnostic assessment:** investigations upon arrival (Table 1) were consistent with a diagnosis of grade 3 SBE complicated by coagulopathy. The thick smear and rapid diagnostic test for malaria were negative. The 20-minute whole blood clotting test (20WBCT) revealed a clotting time of more than 30 minutes. Measurement of fibrinogen and diagnostic imaging were not available.

**Therapeutic interventions:** the patient received two doses of AVS, four units of PRBC, and three units of FFP during hospitalization. He also received ceftriaxone 1 g/24 hours, metronidazole 33 mL/12 hours, and tranexamic acid 400 mg/24 hours. Localized antiseptic treatment was performed on the blisters on the right upper limb.

**Outcome:** unfortunately, the patient died within 1 day of admission from progressive respiratory failure, DIC, and ultimately cardiac arrest.

### Patient 2

**Patient information:** in July 2025, a 12-year-old boy was brought to *L'Abbraccio* Hospital for hemoptysis after being bitten by a snake a few hours prior. The snake was believed to be from the *Viperidae* family. On arrival, he presented with gingival bleeding and edema of the right foot extending to the thigh. Laboratory tests and vital signs were performed (Table 1).

**Clinical findings:** investigations on arrival revealed a prolonged activated partial thromboplastin time of 3.88 minutes. The patient presented with severe anemia (Hb at 5 g/dL). The 20WBCT revealed a prolonged clotting time of 30 minutes. A parasitemia of 622 parasites/μL of *P. falciparum* was detected in the thick smear. Tests for hepatitis B/C and HIV were negative.

**Timeline of current episode:** the edema in the lower limbs gradually spread to the upper limbs. On the third day, the child became pale and agitated, then developed respiratory distress and severe headaches, which were treated with IV morphine 0.02 mg/kg.

**Diagnostic assessment:** a head CT scan could not be performed due to a lack of resources. The diagnosis of grade 3 SBE with coagulopathy was established.

**Therapeutic interventions:** four doses of IPA AVS were administered over two days, along with 60 μg of artesunate at H0, H16, and H24. The patient received six units of FFP, one injection of 250 mg ethamsylate 1u/12h, and two units of PRBC.

**Outcome:** on the fourth day, his neurological condition deteriorated significantly (Glasgow 4/15 E2V1M1), with hypotonia, unresponsive mydriasis, signs of shock, and respiratory failure. The patient died later the same day. The cause of death was severe SBE complicated by suspected subarachnoid hemorrhage and shock.

**Informed consent:** verbal consent for publication was obtained from the parents of these two minor patients. The physician who obtained consent issued a note in the medical files of the two patients confirming parental consent.

## Discussion

The two fatal cases of SBE we describe highlight the severity of illness and suffering caused by SBE in Benin. It is therefore essential to identify the factors contributing to poor clinical outcomes and to implement sustainable solutions. A key factor contributing to suboptimal outcomes is the delay between the snakebite and the start of treatment. In Benin, the average time to consultation for snakebites is between 48 and 72 hours [1]. This delay is often multifactorial. The first patient we presented experienced a delay of over 72 hours before consultation and was referred from an unequipped local hospital. The second was transferred from a rural health center an hour away, reflecting poor access to specialized care. In Benin, cultural beliefs and financial limitations push more than 80% of snakebite victims to opt for traditional remedies as a first option [1]. Such practices are common in several African countries. Crucially, as many bites are not associated with the injection of venom, ineffective treatments may appear to work in a substantial proportion of bites, further perpetuating their use [6].

In our first case, advanced compartment syndrome was highly suspected, but no surgical management followed. Compartment syndrome leads to ischemia, necrosis, and irreversible neuromuscular damage if not promptly treated. The definitive treatment is urgent surgical decompression by fasciotomy, ideally performed within 6 hours of symptom onset, which restores microcirculatory flow and prevents permanent sequelae such as Volkmann's contracture or limb loss [7]. In the context of SBE, however, the decision to perform fasciotomy is especially challenging. Venom-induced edema can mimic compartment syndrome, while concomitant DIC markedly increases the risk of uncontrollable bleeding. In many resource-limited settings, diagnosis relies largely on clinical assessment, as intracompartmental pressure monitoring is often unavailable. Current evidence suggests that fasciotomy should only be performed in cases of SBE complicated by myositis or necrotizing bacterial fasciitis. Moreover, unnecessary fasciotomy has been associated with increased morbidity, including longer hospital stay [4]. In our case, suspected coagulopathy and limited access to monitoring and surgical resources likely influenced clinical management.

The limited availability of SBE epidemiological data in West Africa severely hampers disease control. Current data collection methods in health centers are often inadequate [1]. In order to achieve the WHO's goal of reducing the burden of SBE, it would be necessary to establish a national disease reporting program for SBE, especially in frontline centers in rural areas where most affected people are found. Granting a status of notifiable disease to SBE would enable local health authorities to record cases and identify the most vulnerable communities [4]. Between 2016 and 2018, the WHO Regional Office for Africa implemented a case-by-case notification program for bacterial meningitis cases in Benin as part of the Integrated Disease Surveillance and Response (IDSR) strategy to evaluate the response to the MenAfriVac vaccine. Despite the use of paper forms due to the lack of computerized technology in several centers, the program has proven effective for epidemiological surveillance of bacterial meningitis [8]. It may therefore be feasible to introduce a similar program for SBE in Benin.

Future advances in SBE management must include multiple complementary approaches. Access to effective antivenoms in Africa remains limited due to heavy reliance on imported products that are often poorly matched to local snake species. This problem is exacerbated by a fragile supply chain, marked by the withdrawal of major manufacturers, low global production meeting no more than half of current demand, and high costs restricting accessibility [9]. To ensure antivenoms reach those in need, cold-chain systems and final distribution can be improved through partnerships with programs such as Doctors Without Borders. In parallel, next-generation technologies are being explored to develop more effective antivenoms. One promising approach is the use of recombinant nanobody (VHH)-based antivenoms, which can be engineered to neutralize multiple toxin families across a wide range of snake species. These antibodies can be produced at a lower cost using microbial systems and can prevent both systemic toxicity and local tissue damage, in some cases outperforming conventional plasma-derived antivenoms [10]. This approach offers a promising path toward safer, more effective, and more accessible antivenoms, particularly in regions of Africa where conventional products are often scarce or ineffective.

The poor clinical outcomes observed in the cases we present can be explained by persistent structural barriers, including delays in treatment and a lack of epidemiological surveillance to better equip the most vulnerable communities. Our study highlights these systemic barriers in resource-limited countries such as Benin and suggests possible solutions to reduce mortality. There are some limitations to our study. Biological tests to confirm coagulopathy are incomplete due to a lack of diagnostic modalities. In both cases, no imaging was performed to confirm the suspected complications. Furthermore, accurate identification of the snake species involved could not be performed, limiting the specificity of the diagnosis.

## Conclusion

Our case report presents the known hemorrhagic effects of *Viperidae* venom and identifies the various factors limiting optimal medical treatment in cases of SBE. It also highlights the need to implement public health programs aimed at improving the availability of epidemiological data and access to appropriate medical care for cases of SBE in sub-Saharan Africa.
